# Visually Evoked Postural Responses (VEPRs) in Children with Vestibular Migraine

**DOI:** 10.3390/children9010014

**Published:** 2021-12-27

**Authors:** Riccardo Nocini, Carlo Baraldi, Enrico Apa, Andrea Ciorba, Daniele Monzani, Silvia Palma

**Affiliations:** 1 Section of Ears, Nose and Throat (ENT), Department of Surgical Sciences, Dentistry, Genecology and Pediatrics, University of Verona, 37100 Verona, Italy; Riccardo-nocini@univr.it; 2 School of Pharmacology and Clinical Toxicology, Department of Biomedical, Metabolic and Neural Sciences, University of Modena and Reggio Emilia, Via del Pozzo 71, 41124 Modena, Italy; claudio.baraldi@unimore.it; 3 Otolyngology Unit, University of Modena and Reggio Emilia, Via del Pozzo 71, 41124 Modena, Italy; enrico.apa@unimore.it (E.A.); daniele.monzani@unimore.it (D.M.); 4 Department of Neurosciences and Rehabilitation, ENT Clinic, University of Ferrara, Via Aldo Moro 8, 44124 Ferrara, Italy; andea.ciorba@unife.it; 5 Audiology, Primary Care Department, Via del Pozzo 69, 41100 Modena, Italy

**Keywords:** migraine, optokinetic stimulation, posturography, visual–vestibular mismatch, nystagmus

## Abstract

Vestibular migraine (VM) is the most common cause of episodic vertigo in children. Vertigo, nausea, dizziness and unsteadiness are often complained of by children with migraine, which can precede, follow or be present simultaneously with headache. The aim of this study was to use posturography to investigate the visually evoked postural responses (VEPRs) of children with VM and compare them to data obtained from children with primary headache (M) and controls (C). Twenty children diagnosed as affected by VM, nineteen children with M without aura and twenty healthy subjects were recruited in this cross-sectional study. Posturography was performed by a standardized stabilometric force-platform (Svep-Politecnica) in the following conditions: open eyes (OE), closed eyes (CE) and during full-field horizontal optokinetic stimulation (OKN-S). Electronystagmography was performed simultaneously to analyze optokinetic reflex parameters. In the OE condition, no difference was found between groups with respect to body sway area. In contrast, this parameter increased in the two pathological groups with respect to controls in the CE condition. The optokinetic stimulations also induced a similar increase of body sway area in the M group relative to controls, but a further increase was elicited in the VM group. Electronystagmographic recording also revealed different optokinetic reflex parameters in the latter groups. This study disclosed an abnormal sensitivity of children with M and VM to full-field moving scenes and a consequent destabilization of posture, as documented by the abnormal VEPRs. Children with VM were particularly exposed to this risk. Possible clinical implications of these findings are discussed.

## 1. Introduction

Episodic vertigo in children is common but it frequently remains an underestimated symptom despite the fact that it could be detrimental to regular school attendance and leisure activities [1]. The most common cause of episodic vertigo in children is vestibular migraine (VM) [2], and headache can occur before, simultaneously or after vestibular symptoms; phonophobia, photophobia and motion sickness are often associated complaints.

The mechanisms that account for vestibular symptoms in adults with VM have been extensively investigated and a dysfunctional vestibulo–thalamo–cortical network [3], the involvement of subclinical cerebellar–vestibular pathways [4] and structural changes in the central vestibular cortex [5,6] have been proposed at different times. The debate over the mechanistic basis of VM in childhood is still open. Higher genetic susceptibility is suspected, given that most children with VM have a family history of migraines, but evidence to support heritability is limited [7]. Once a correct diagnosis is established, medications are frequently effective in reducing symptoms [8]. A careful examination of trigger symptoms is a prerequisite for successful non-pharmacological prophylaxis and rehabilitation [9].

It is known that sensitivity to repeated, moving full-field visual scenes and motion is frequently reported in children with vestibular disorders and migraine [10]. A previous case–control study in children with migraine without vestibular symptoms suggested abnormal visually-evoked postural responses (VEPRs) with respect to healthy subjects when exposed to a full-field horizontal optokinetic stimulation (OKN-S) [11]. In addition, adult patients with both migraine (M) and VM exhibited a greater instability induced by OKN-S in the interictal period than healthy subjects [12,13]. Furman et al. stated that balance perturbation induced by OKN-S was greater in VM patients compared to subjects with M and suggested that this difference depends on a greater susceptibility of the former when exposed to large, moving scenes, such as traffic, crowded supermarkets, etc. [14].

Dizziness and unsteadiness induced by optic flow, the so-called visually induced dizziness (ViD), is thought to depend on the inability of the central nervous system to process “conflicting” information from the vestibular and visual systems, such as those elicited in humans by standing still on the sidewalk and watching cars passing by. In such a situation, signals from the visual system suggest motion in contrast to vestibular and somatosensory information that indicates no head and body movement (visual–vestibular mismatch). Since ViD and balance disturbances can be improved by rehabilitation techniques that are based on OKN-S [15], it is reasonable to suppose that children with VM could benefit from this approach. Unfortunately, there is currently no systematic study of VEPRs in this population, and it is not yet known if visual optic flow is an effective trigger of balance perturbation. Therefore, the aim of this study was to analyse VEPRs in children affected by VM.

## 2. Materials and Methods

This cross-sectional study was based on children referred to the Tertiary Centre for Vestibular and Balance Disorders of the University Hospital of Modena from the Centre for the Diagnosis and Treatment of Headaches of the same institution between 2013 and 2019.

The casuistry was composed of school-age children (6–12 years old) affected by vestibular migraine (group VM) and by migraine without aura (group M). Diagnosis has been defined according to the criteria proposed for adults recently reintroduced by the Committee for the Classification of Vestibular Disorders of the Bárány Society and the International Headache Society to define VM in childhood [16,17,18] 

Diagnostic criteria for VM were: at least 5 episodes with vestibular symptoms lasting between five minutes and 72 h; a history of migraine with\without aura; at least half of the episodes associated at with one of the following three migraine features: headache, (with at least two of the following four characteristics: One-sided location; Pulsating quality; Moderate or severe pain intensity; Aggravation by routine physical activity) photophobia and phonophobia; visual aura.

Children with middle and/or inner ear diseases were excluded. Parents were also asked to complete the Child Behaviour Checklist [19] to rule out subclinical psychological disorders, such as anxiety, that could account for an impairment of vestibulo–spinal reflexes during OKN stimulation [20]; subjects with psychological distress were excluded.

A group of children, well-matched for sex and age, selected from a database of healthy children, served as controls (group C).

### 2.1. Procedures

#### Preliminary Vestibular Tests

Subjects admitted to the study underwent a battery of preliminary vestibular tests, including videonystagmoscopy, electronystagmography with caloric tests (no specific test was performed to analyse pursuit gain and saccadic accuracy) and cervical vestibular evoked potentials (cVEMPs).

The tests were performed during symptom-free periods in order to detect spontaneous, positional and paroxysmal nystagmus and peripheral vestibular hyporeflexia/areflexia.

Nystagmus was considered pathological if the slow-phase velocity was greater than 3°/section in primary gaze. Caloric tests, performed according to the Fitzgerald–Hallpike technique, were considered pathological if the Jongkees’s formula was greater than 25%.

cVEMPs were considered abnormal if absent or with increased peak latencies or with large interaural amplitude asymmetry. Subsequently Children were Submitted to the Stabilometric Session

### 2.2. Stabilometric Session

**Figure 1:**
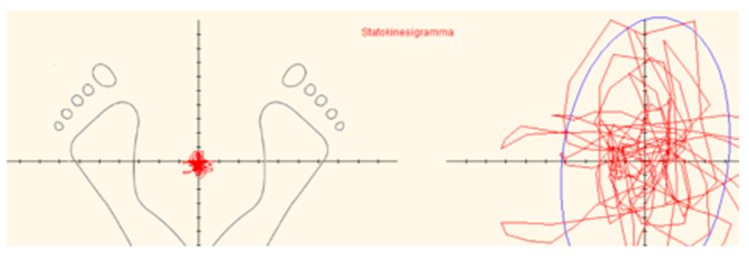
Statokinesigram (SKG). SKG surface (SKG-S) is expressed in square millimeters (mm^2^) and was computed by calculating an elliptic area which corresponds to 90% of the COP positions over time. This procedure is designed to eliminate 10% of the more extreme positions of COP, which could be due to involuntary perturbations of quiet stance.

Static posturography was performed by a standardized stabilometric force platform (Svep-Politecnica). Technical data and procedure details are described in previous studies [11,20]. Briefly, the displacements of the centre of pressure (COP) that roughly correspond to the projection of the body centre of gravity to the ground were continuously registered during a period of 52 s with a sampling frequency of 50 Hz, and the resulting sway path, the so-called statokinesigram (SKG), was recorded (Figure 1).

Four SKGs were obtained from all subjects while standing still on the stabilometric platform, under different visual conditions:

(1) With open eyes (OE) while gazing a vertical bar at a distance of 150 cm (cm) in front of them;

(2) Closed eyes (CE);

(3–4) During a full-field horizontal OKN-S that was delivered onto the wall in front of them at a 2 m distance, it was represented by vertical alternating white and dark bars rolling at a regular speed of 30° per second, from left to right and vice versa.

The sequence of the tests was randomly changed from one child to another, to avoid adaptation due to a possible learning effect.

The ratio of SKG-S measured under CE and OE conditions multiplied per 100, the so-called ‘stabilometric Romberg quotient’ (RI), was computed to assess the influence of static visual cues on the multisensory control of posture. Furthermore, the ratio between the sum of SKG-S recorded in the two tests during OKN-S and SKG-S in the OE condition was computed (OKN destabilizing index). This ratio is indicative of each subject’s sensitivity to moving visual surroundings with respect to static visual references [21].

### 2.3. Electronystagmography Recordings

Electronystagmography was performed during the last two tests in order to record OKN reflex parameters: angular slow phase velocity (ASPV), number of saccades (NS), mean peak velocity of saccades (MPVS). ASPV and MPVS are expressed in degrees/second. Electronystagmographic recordings of OKN reflexes were executed over a period of 20 s (excluding the beginning and the final phase of the stimulation).

### 2.4. Statistical Analysis

Continuous variables were expressed as mean ± standard deviations (SD); continuous variables were compared between three groups (M, VM and C) with the one-way analysis of variance followed by the Tukey–Kramer post hoc comparison test. Fisher’s exact test was used to analyse gender homogeneity between patients and controls. *p*-values lower than 0.05 were considered significant. Statistical Package Software, version 16, was used for statistical analyses.

## 3. Results

Demographic characteristics of subjects and headache features are indicated in Table 1.

The VM group was composed of 20 children, the M group of 19 and in the C group there were 20 children. Gender and age distribution among M, VM and C groups were not different. At this point, the participants of the groups were considered well-matched.

Furthermore, no difference was found between the VM and M groups with respect to the mean duration of illness and the time interval between the last headache attack and the day of examination. In no case was this interval shorter than 9 days or longer than 12 days with respect to the vertigo crisis. In all VM cases, headache was pulsatile and had one-side location features. In the M group, headache was unilateral in 15 children, pulsatile in 14. The number of headache attacks per month was lower in the VM than in the M group.

Concerning vestibular symptoms (VM group), in five subjects they preceded the crises (precritical), in eight subjects they accompanied the crises (critical) and in seven subjects they followed them (postcritical).

None of the participants developed headache during or after the stabilometric session, while nearly one half of children (nine cases) with VM complained of oncoming nausea and imbalance during OKN-S. Nevertheless, no subject had to discontinue either test.

### 3.1. Stabilometric Data

Stabilometric data are summarized in Table 2, where the one-way analysis of variance and the Tukey–Kramer post hoc tests are reported. No significant difference between groups was observed concerning the value of SKG-S under the OE condition.

*In the CE condition*, SKG-S was significantly increased in both VM and M children compared to the C group, with no difference between the former two.

*Stabilometric RI* was increased only in the VM group with respect to healthy subjects, with no difference between the two pathological samples.

*SKG-S induced by OKN-S to the right* evidenced differences between groups: the M group did not exhibit an increased SKG-S with respect to controls, while a significant difference was shown between VM and M children and between the former and healthy controls.

*SKG-S recorded with OKN-S to the left* were different between groups. The tests did not induce statistically different SGK-S between healthy subjects and the M group. However, the VM group exhibited increased SKG-S with respect to both M and C children. As a consequence, the OKN destabilizing index was greater in the VM group compared to healthy subjects and M children, and no difference was evident between the latter two.

### 3.2. Electronystagmographic Recordings during Stabilometric Session

Electronystagmographic results are summarized in Table 3 where one-way analyses of variance and Tukey–Kramer post hoc tests are reported.

Electronystagmographic recordings of OKN reflexes induced by horizontal optic flow delivered *to the right* revealed a reduced ASPV in VM with respect to both M and C groups, and no difference was found between M and C subjects. Total NS was also lower in the VM group than in M children and in controls it was found to be intermediate between the latter two. MPVS was reduced in VM with respect to both M and C groups, and no difference was revealed between these two latter groups.

Similarly, the optic flow delivered *to the left* induced a reduced ASPV in VM with respect to both M and C groups, and no difference was recorded between M and C subjects.

In the same session, NS was accordingly reduced in VM with respect to both M and C groups, and no difference was elicited between the latter two.

Finally, MPVS showed a significant reduction in children with VM compared to M children and healthy controls. This OKN reflex parameter did not significantly vary between M and C groups.

## 4. Discussion

This study investigated the postural control of children affected by migraine without aura and by VM in a juvenile population. In addition to base stabilometric conditions by static posturography, two further trials with horizontal OKN-S were performed to explore VEPRs.

A preliminary vestibular examination excluded the presence of peripheral vestibular disorders that could account for postural perturbations per se during both quiet stance and, above all, during OKN-S [22]. At this stage, subjects with pathological ocular signs (spontaneous nystagmus and paroxysmal and persistent positional nystagmus), hyporeflexia as determined by caloric tests and altered vestibulo-collic reflexes were not included in this casuistry, in contrast to other studies on both children and adults [23,24,25]. Postural stability in the OE condition was not different among groups, in contrast to CE, which disclosed a significant increase of SKG-S both in M and VM groups with respect to controls. Accordingly, stabilometric RI was increased in the former groups. It is therefore suggested that both M and VM in childhood entail a perturbation of the postural system due to a central vestibular dysfunction rather than a peripheral one. However, if static visual references are available, these postural perturbations become negligible. It is well-known that, among all sensory systems of the body, humans primarily use vision to enable the brain to integrate multisensory information regarding the relative position of the body in space and continuously adjust posture accordingly [26]. Moreover, when vestibular information is defective, the central nervous system compensates for sensory deficiencies by further increasing the weight of visual cues to preserve upright posture [27]. The reliance on static visual cues for postural control, so-called ‘visual dependency’, is not only present in adults with vestibular disorders [28] but also in healthy children under 16 years and adolescents [29]. This study documented the increase of ‘visual dependency’ in children with VM in accordance with previous studies of children with M [11] and adults with VM [13]. This study, however, showed that dynamic visual cues, such as those generated by OKN-S, are responsible for a destabilization of posture significantly greater in the two pathological groups than in controls and documented a further increase in children with VM relative to those with M.

Since these differences were present regardless of the direction of the OKN-S, the OKN destabilizing index behaved in accordance. A possible explanation for the difference of VEPRs between M and VM groups could be suggested by the difference of OKN reflex parameters recorded during optic flow with particular regard to ASPV and MPVS. It is generally accepted that the slow and fast phases of the OKN reflex consist of ocular pursuit and saccades, respectively, and abnormalities in both of these have been reported in children with vestibular migraine perhaps due to a central vestibular dysfunction [25]. The increase of postural instability in children with M and VM in response to OKN-S recalls previous results both in children [11] and adults [12,13,14]. The exact mechanism of these abnormal VEPRs is not known bus some experiments suggested an altered interaction between visual and vestibular cortical networks [12,30]. It was also shown that healthy children may be more prone to ViD than adults because their ability to maintain adequate postural control in response to multisensory contradictory information is not complete before adolescence [31]. Taken together, it is reasonable to suggest that large moving visual scenes, such as those commonly encountered in traffic or supermarkets, could be regarded as a trigger of balance disturbance in children with M and VM, even in vertigo- and pain-free intervals. This result closely resembles that of a recent investigation into adults with VM [32]. This study concluded that recurrent attacks of dizziness are induced by routine moving visual stimuli in a high percentage of cases, so that visually busy environments can be considered as triggers.

The stabilometric results during optic flow reached an excellent significance level, so that the different behaviour of VEPRs in children suffering from vestibular migraine and in healthy controls has definitely been assessed.

The main limitation of the study is the small sample size, primarily due to the strict inclusion criteria. It should also be observed that electronystamographic tests for latency and the accuracy of horizontal and vertical saccades and pursuit gain were not executed in this study, so that the involvement of these ocular motor systems has only indirectly been analysed.

## 5. Conclusions

Postural strategies of children with migraine are different to those of healthy subjects, similarly to adults. These differences are even more relevant in children diagnosed as affected by VM. The stabilometric results of this study indicate a bimodal processing of visual information in the pain- and vertigo-free intervals: static visual references are effective in reducing body sway, and, in contrast, moving visual cues, such as those generated by OKN-S, are detrimental to postural control and a remarkable increase of body sway is clearly documented. This destabilization of posture is independent of the presence of peripheral vestibular disorders, and a reduced ability of the central nervous system to resolve the visual–vestibular mismatch induced by optic flow while standing still is postulated. For the first time, this study has shown that children with VM are critically destabilized by moving visual stimuli in the interictal period and this behavior could be interpreted as a marker of the disease. Finally, further studies are needed to verify if rehabilitation programs that incorporate OKN-S for balance disorders and motion sickness could be beneficial also for children with VM.

## Figures and Tables

**Table 1:** Demographic characteristics and headache features.

	C	VM	M	*p*-Value *
Number of subjects	20	20	19	
Males	10 (50%)	9 (45%)	9 (47.4%)	0.499
Age (years)	10.1 ± 1.5	8.9 ± 1.7	9.6 ± 1.6	0.105
Headache length (months)		13.4 ± 4.3	12.0 ± 5.1	0.516
One side pain		20 (100%)	15 (79%)	0.047
Pulsating pain		20 (100%)	14 (73.7%)	0.020
Average time interval from the last headache attack and examination (days)		16.9 ± 4.7	14.0 ± 2.7	0.063
Number of headache attacks per month		1.8 ± 1.2	2.8 ± 1.2	0.012

* *p*-values refer to one-way analysis of variance (followed by Tukey–Kramer post hoc test for age). It refers to Fisher’s exact test for gender.

**Table 2:** Stabilometric parameters; mean value, standard deviation, confidence interval (95%) with lower and upper bounds, are reported for each group.

	Groups	Mean	SD	Confidence Interval for the Mean (95%)		Difference between 3 Groups *^,^**	*p*-Value
				Lower Bound	Upper Bound		
SKG-S (mm^2^), OE	C	284.3	133.4	219.8	348.6	C versus M	0.565
	M	339.7	195.4	245.5	433.9	M versus V M	0.436
	VM	405.9	166.4	328.1	483.8	V M versus C	0.668
	Total	344.4	171.6	299.3	389.5	between Groups	0.840
SKG-S (mm^2^), CE	C	272.2	116.8	215.9	328.5	C versus M	0.049 *
	M	437.0	204.4	341.8	532.1	M versus V M	0.124
	VM	575.4	294.1	437.8	713.1	V M versus C	<0.001 *
	Total	430.1	247.3	365.7	495.8	between Groups	<0.001 **
R I	C	103.1	31.2	88.1	118.1	C versus M	0.087
	M	142.2	56.7	13.1	114.9	M versus V M	0.678
	VM	154.6	70.9	121.4	187.8	V M versus C	0.015 *
	Total	133.7	56.0	118.1	149.2	between Groups	0.016 **
SKG-S (mm^2^); OKN right	C	326.5	123.1	207.8	326.4	C versus M	0.088
	M	456.1	391.4	267.4	326.5	M versus V M	0.006 *
	VM	737.2	227.4	630.8	843.7	V M versus C	<0.001 *
	Total	491.1	329.8	404.4	577.9	between Groups	<0.001 **
SKG-S (mm^2^); OKN left	C	260.2	161.2	182.5	337.8	C versus M	0,146
	M	436.3	340.9	272.0	600.6	M versus V M	<0.001 *
	VM	833.1	315.6	685.4	980.7	V M versus C	<0.001 *
	Total	515.4	370.5	418.0	612.8	between Groups	<0.001 **
OKN destabilising index	C	2.1	0.9	1.6	2.5	C versus M	0.459
	M	2.6	1.1	2.1	3.1	M versus V M	0.001 *
	VM	4.5	2.2	3.5	5.5	V M versus C	<0.001 *
	Total	3.1	1.8	2.6	3.6	between Groups	<0.001 **

Significant *p*-values refer to one-way analyses of variance (**) and Tukey-Kramer post-hoc (*). C = controls, M = children with migraine, VM = children with vestibular migraine. SKG-S = statokinesigram, OE = open eye, CE = closed eye.

**Table 3:** Optokinetic reflexes parameters recorded by electronystagmography during the two stabilometric tests performed with optokinetic horizontal stimulations.

	Groups	Mean	SD	Confidence Interval (95%) for the Mean		Difference between 3 Groups *^,^**	*p*-Value
				Lower Bound	Upper Bound		
ASPV (OKN right)	C	12.9	0.6	12.6	13,2	C versus M	0.996
	M	12.9	0.7	12.6	13.3	M versus VM	<0.001 *
	VM	11.4	1.7	10.6	12.2	V M versus C	<0.001 *
	Total	12.4	1.3	12.1	12.8	between Groups	<0.001 **
NS (OKN right)	C	24.5	24.5	23.3	25.7	C versus M	0.780
	M	24.0	2.2	23.0	25.0	M versus VM	0.048 *
	VM	22.3	1.9	21.5	23.2	V M versus C	0.010 *
	Total	23.6	2.3	23.0	24.2	between Groups	0.009 **
MPV (OKN right)	C	325.4	9.7	320.7	330.0	C versus M	0.224
	M	319.5	10.8	314.2	324.7	M versus VM	0.030 *
	VM	310.4	11.8	304.8	315.9	V M versus C	<0.001 *
	Total	318.2	12.4	315.0	321.5	between Groups	<0.001 **
ASPV (OKN left)	C	12.7	0.5	12.5	13.0	C versus M	0.997
	M	12.8	0.7	12.5	13.1	M versus VM	0.001 *
	VM	11.6	1.5	10.8	12.3	V M versus C	0.002 *
	Total	12.3	1.2	12.0	12.7	between Groups	<0.001 **
NS (OKN left)	C	24.7	2.6	23.0	25.5	C versus M	0.901
	M	23.9	2.1	22.9	24.9	M versus VM	0.002 *
	VM	21.3	3.0	19.8	22.6	V M versus C	0.007 *
	Total	23.1	2.9	22.3	23.7	between Groups	0.001 **
MPV (OKN left)	C	322.2	7.6	318.6	325.8	C versus M	0.998
	M	322.7	11.9	317.0	328.5	M versus VM	0.002 *
	VM	309.7	12.8	303.8	315.7	V M versus C	0.002 *
	Total	318.1	12.4	314.8	321.3	between Groups	0.001 **

Values are reported as means and standard deviations. Confidence interval (95%), lower and upper bounds are also reported. Significant P values refer to one-way analyses of variance (**) and Tukey-Kramer post-hoc (*). C = controls, M = children with migraine, VM = children with vestibular migraine of childhood. ASPV = angular slow phase velocity, NS = number of saccades, MPV = mean peak velocity of saccades.

## Data Availability

The data presented in this study are available from the corresponding author on request due to privacy restrictions.

## Abbreviations

C	Control
COP	Centre of pressure
CE	Closed eyes
OE	Open eyes
M	Migraine
VM	Vestibular migraine
VMC	Vestibular migraine in childhood
OKN	Optokinetic
RI	Romberg’s index
SKG	Statokinesigram
SKG-S	Statokinesiogram surface
ASPV	Angular slow phase velocity
MPVS	Mean peak velocity of saccades
VEPR	Visually evoked postural response
ViD	Visuall -induced Dizziness
cVEMPs	Cervical vestibular evoked potentials
Centimeters	cm
Millimeters	mm
Square millimeters	mm^2^
